# Corrigendum to “Perinatal care and breastfeeding education during the COVID‐19 pandemic: Perspectives from Kenyan mothers and healthcare workers”

**DOI:** 10.1111/mcn.13679

**Published:** 2024-06-07

Ickes S. B., Lemein H., Arensen K., Kinyua J., Denno D. M., Sanders H. K., Walson J. L., Martin S. L., Nduati R., & Palmquist A. E. L. (2023). Perinatal care and breastfeeding education during the COVID‐19 pandemic: Perspectives from Kenyan mothers and healthcare workers. *Maternal & Child Nutrition*, 19(4), e13500. doi:10.1111/mcn.13500. Epub 2023 May 19.

Lucy Browning was accidentally omitted from the list of contributing authors. Lucy Browning should be listed as the sixth author for this manuscript.

We apologize for this error.

